# Robust and ultralow-energy-threshold ignition of a lean mixture by an ultrashort-pulsed laser in the filamentation regime

**DOI:** 10.1038/s41377-021-00496-8

**Published:** 2021-03-05

**Authors:** Hongwei Zang, Helong Li, Wei Zhang, Yao Fu, Shanming Chen, Huailiang Xu, Ruxin Li

**Affiliations:** 1 State Key Laboratory of Integrated Optoelectronics, College of Electronic Science and Engineering, Jilin University, 130012 Changchun, China; 2 Institute of Atomic and Molecular Physics, Jilin University, 130012 Changchun, China; 3 CAS Center for Excellence in Ultra-intense Laser Science, 201800 Shanghai, China; 4 State Key Laboratory of High Field Laser Physics, Shanghai Institute of Optics and Fine Mechanics, Chinese Academy of Sciences, 201800 Shanghai, China; 5 School of Physical Science and Technology, ShanghaiTech University, 201210 Shanghai, China

**Keywords:** Nonlinear optics, Optical techniques

## Abstract

Laser ignition (LI) allows for precise manipulation of ignition timing and location and is promising for green combustion of automobile and rocket engines and aero-turbines under lean-fuel conditions with improved emission efficiency; however, achieving completely effective and reliable ignition is still a challenge. Here, we report the realization of igniting a lean methane/air mixture with a 100% success rate by an ultrashort femtosecond laser, which has long been regarded as an unsuitable fuel ignition source. We demonstrate that the minimum ignition energy can decrease to the sub-mJ level depending on the laser filamentation formation, and reveal that the resultant early OH radical yield significantly increases as the laser energy reaches the ignition threshold, showing a clear boundary for misfire and fire cases. Potential mechanisms for robust ultrashort LI are the filamentation-induced heating effect followed by exothermal chemical reactions, in combination with the line ignition effect along the filament. Our results pave the way toward robust and efficient ignition of lean-fuel engines by ultrashort-pulsed lasers.

## Introduction

Laser ignition (LI) is a promising electrode-less alternative to electronic spark ignition of lean-fuel/air mixtures, offering high thermal efficiency with low harmful emissions^1^. One of the most widely adopted LI methods is nanosecond laser-induced spark ignition (ns-LISI)^2–7^, in which combustible mixtures undergo multiphoton ionization followed by avalanche breakdown, resulting in high-temperature and high-pressure plasma along with shockwaves. After shockwave expansion, the hot plasma consisting of many atoms and ions cools, and evolves to the flame kernel, finally developing complete combustion through chemical branching reactions. However, inevitable shot-to-shot energy fluctuations resulting from ns light sources lead to the stochastic nature of the breakdown, influencing reaction routes and producing potential misfiring^2^.

Although LI is not a new concept, it is commonly deemed that igniting lean-fuel mixtures by an ultrashort femtosecond (fs) laser is hard to realize, since avalanche breakdown cannot occur on the fs timescale, and the fs-laser-induced plasma temperature is 1–2 orders of magnitude smaller than that pumped by ns lasers^8,9^, both of which decrease the lean-fuel ignitability. Indeed, researchers have failed to ignite lean mixtures using intense fs-laser-induced plasma sparks in a tight focusing scheme^10^. Alternatively, it was suggested that a fs laser can be an auxiliary source to assist the plasma formation and successive flow control in ns-LISI^10^, as well as to enhance the combustion speed and stability of flames when its repetition rate is high (*≥*500 Hz)^11,12^.

Here, we report the unexpected results of proof-of-principle lean-fuel combustion with a high rate of success, using an intense fs laser. Rather than relying on a very tightly focused fs laser beam, we employed an intense fs laser propagating in a lean methane/air mixture in the self-channeling regime popularly termed fs laser filamentation^13,14^. The dynamic equilibrium between self-focusing and plasma defocusing in the laser filament allows for the generation of several Rayleigh range or longer plasma channels with the laser intensity clamped at the ∼50–100 TW cm^−2^ level. Recent studies have revealed that fuel molecules can be activated and even fragmented by high-intensity laser filaments, producing many combustion intermediates^15^. In particular, the long filament provides the possibility of “multipoint” ignition along the filament, hereafter referred to as “line” ignition, which may improve the ignition reliability of lean mixtures^7^. In addition, inside the fs laser filament, although the initial temperature of gas molecules determined through various energy deposition pathways, such as multiphoton/tunnel ionization, dissociation, Raman excitation, and collision excitation^16–21^, is only ~1400 K (ref. ^16^), the low-temperature oxidation reaction of methane molecules can still occur^22^, which may allow for the initiation of combustible chemical reactions.

In the present study, we demonstrated the realization and robustness of fs LI by irradiating a lean methane/air mixture with an intense 40-fs, 800-nm laser pulse in the filamentation regime. We reveal that the pump laser energy for lean combustion can decrease to ∼1.5 mJ with an energy deposition of ∼25%, implying that it takes only sub-mJ energy to achieve fs LI. By recording time-resolved flame kernel images and optical emission spectroscopy (OES) spectra at different pump laser energies, we further show that the resultant OH radical yield plays an essential role in lean methane/air combustion, which dramatically increases as the pump laser energy reaches the minimum ignition energy. We ascribe the ultrashort LI mechanisms to the thermal effect by laser energy deposition in the filament followed by combustion chemical reactions and the robustness to the line ignition effect.

## Ultrashort laser ignition at different energies

**Fig. 1:**
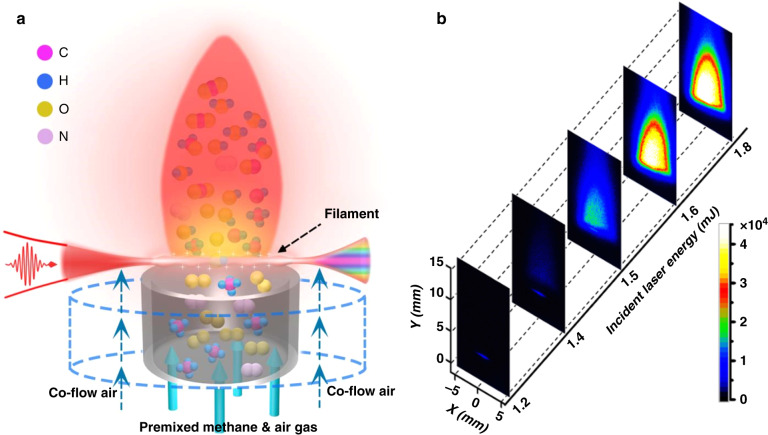
Laser filament ignition concept and ultralow-energy-threshold ignition images. **a** Schematic diagram of the ultrashort laser filament ignition of a premixed lean methane/air mixture flow. **b** Side-view images of the methane/air mixture flow irradiated by an intense fs laser filament at different incident laser energies

With the experimental setup shown in Fig. 1a (for experimental details, see “Methods”), in Fig. 1b, we show the experimentally recorded side-view images of the laminar premixed methane/air flow irradiated by the intense fs laser filaments at different input laser energies, all of which are higher than the critical power for self-focusing^23^. It can be seen from Fig. 1b that when the input laser energy was 1.2 mJ, except for the fs-filament-induced fluorescence along the filament, no flame could be observed; that is, LI failed under this condition. As the laser energy increased to 1.4 mJ, a weak flame above the filament started to appear, and as the laser energy further increased, a flame with strong optical emission could be observed, which blurred the filament-induced fluorescence. The above results clearly indicate that ultrashort LI can be unambiguously achieved in the lean methane/air mixture when the input laser energy is >1.5 mJ, which is estimated to be one order of magnitude lower than that (several tens of mJ) in the ns-LISI scheme^2,9,24^. In addition, in the fs filament ignition, we obtained a lean limit of *φ* = 0.75 (*φ*: equivalence ratio of fuel to air) when the laser energy was set at 1.8 mJ, which was also approximately one order of magnitude smaller than that (several tens of mJ) in ns-LISI for the same lean limit^24^. It should be emphasized that we tested the LI at *φ* = 0.82 with a 1.8-mJ laser energy >1000 times and consequently achieved a 100% rate of success, showing the robustness of this approach for igniting lean mixtures.

## Dynamic evolution of the flame kernel

**Fig. 2:**
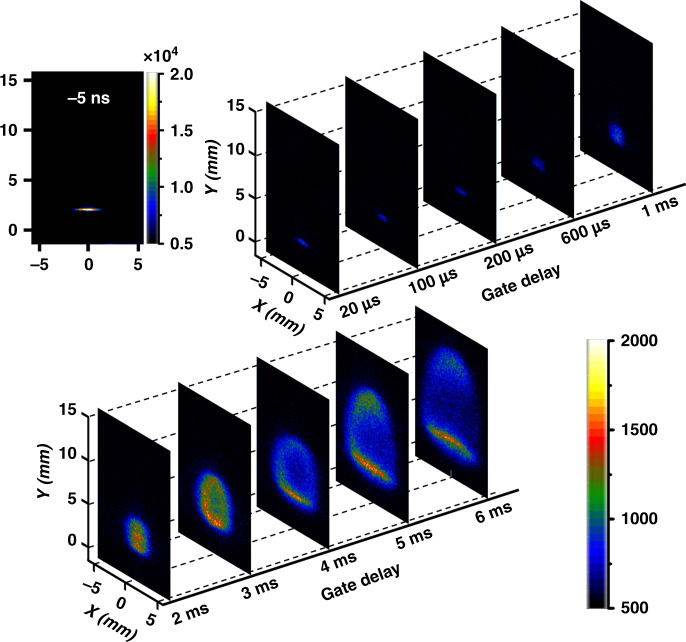
Temporal ignition evolution. Side-view images for the dynamic evolution of the flame kernel ignited by the fs laser filament

To determine the dynamics of laser filament ignition, we show, in Fig. 2, the recorded side-view images of the lean-fuel flow pumped by 1.8-mJ input laser energy with different ICCD time delays at a fixed time window of 50 μs (for experimental details, see “Methods”). As shown in Fig. 2, the temporal evolution of the flame kernel to the propagating flame can be clearly observed, in which strong optical emissions from the plasma filament are seen at the interaction time window (*t* = −5 ns), and then a weak flame kernel forms at the time delay of *t* = 20 μs. After generation, the flame kernel vertically expands outward with the filament axis. As the time delay further increases, the upper and lower fronts of the flame kernel fronts propagate along the opposite directions, and the flame kernel evolves to a spherical shape at *t* ∼1 ms. When the delay time increases, the laser-induced flame appears clearly, which further evolves to a large size. In addition, as combustion develops, the upper and lower fronts of the flame behave differently, having cone-shaped and nearly flat structures, respectively, similar to those in ns-LISI^25^.

**Fig. 3:**
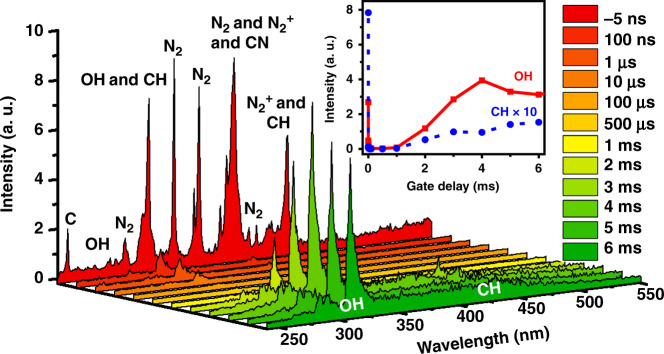
Time-resolved OES spectra. Filament-induced OES spectra of the lean-fuel mixture obtained with different temporal delays of the ICCD. Inset: the signal intensities of OH (red rectangle) and CH (blue dot) radicals measured as a function of the gate delay

Moreover, we measured time-resolved OES spectra to investigate the mechanisms of flame formation, as shown in Fig. 3. For the gate delay of *t* = −5 ns, multiple spectral bands appear in the OES spectrum, which are assigned to the fluorescing free radicals of CH (431.4 nm: A^2^Δ-X^2^Π; 314.5 nm: C^2^Σ-X^2^Π) and OH (289.2 and 308.9 nm: A^2^Σ^+^-X^2^Π^+^), and the neutral and ionic nitrogen molecules of N_2_ (C^3^П_*u*_-B^3^П_*g*_) and N_2_^+^ (B^2^Σ_*u*_^+^-X^2^Σ_*g*_^+^)^26,27^. When the time delay increases, except for the OH radicals, the fluorescence intensities of other species dramatically decrease because these species are all short lived, usually approximately a few tens of nanoseconds or shorter in a laser filament^28^. The OH fluorescence intensity decreases slowly at a microsecond timescale, implying that it may result from multiple physical processes, including competition between the production and consumption of OH radicals, as well as flame kernel expansion after the shockwave. When the delay time exceeds 1 ms, the high-intensity fluorescence emissions of OH and CH radicals at ~308.9 and 431.4 nm reappear in the OES spectra due to the propagating flame^29^, indicating that the methane/air flame appears with an ignition delay time of ~1 ms.

## Energy deposition measurement

**Fig. 4:**
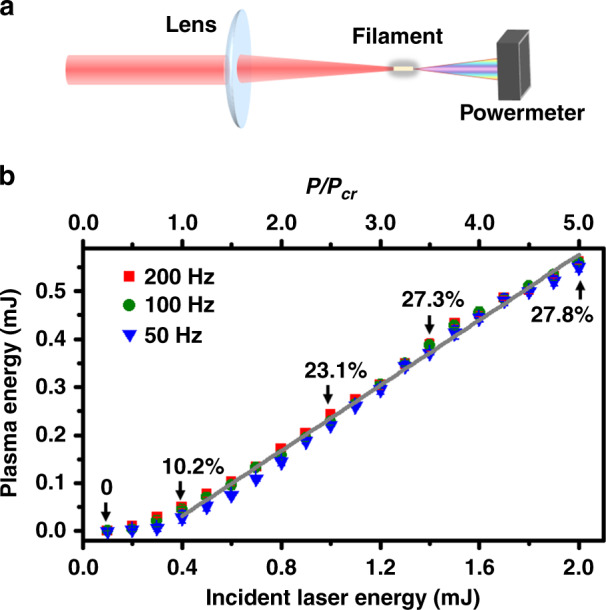
Energy deposition measurement. **a** Schematic of the experimental setup for measuring energy deposition. **b** Plasma energy couplings in the premixed methane–air flow measured with laser repetition rates of 50 (blue triangle), 100 (green circle), and 200 Hz (red square)

To explore the mechanism responsible for the filament-induced ignition, we also investigate the energy deposition of the filamentation pulse into the lean-fuel methane/air flow by the experimental setup shown in Fig. 4a (see “Methods” for details). Figure 4b shows the dependence of the coupled-to-plasma energy (CPE) on the input laser energy at different laser repetition rates. It can be seen in Fig. 4b that the CPE increases linearly as the input laser energy increases from 0.4 to 2.0 mJ, which is ascribed to the linear dependence of the filament plasma volume on the input laser energy^30^. As the incident laser energy is in the range of 0.1–0.4 mJ, the CPE is very low and even reaches zero due to the reduced plasma generation efficiency. It can also be seen from Fig. 4b that all the measured energy deposition efficiencies are <30%, which are much lower than those (40–60%) of ns-LISI^25^. Clearly, the minimum ignition energy can decrease to the sub-mJ level (<0.4 mJ), which is approximately one order of magnitude smaller than the reported values in ns-LISI^6^. Moreover, it is found that the CPE efficiencies are nearly the same in the range for different laser repetition rates, indicating that the energy transfer from the laser pulse to the plasma is insensitive to the ignition and combustion processes.

## OH radical fluorescence at the early time stage

**Fig. 5:**
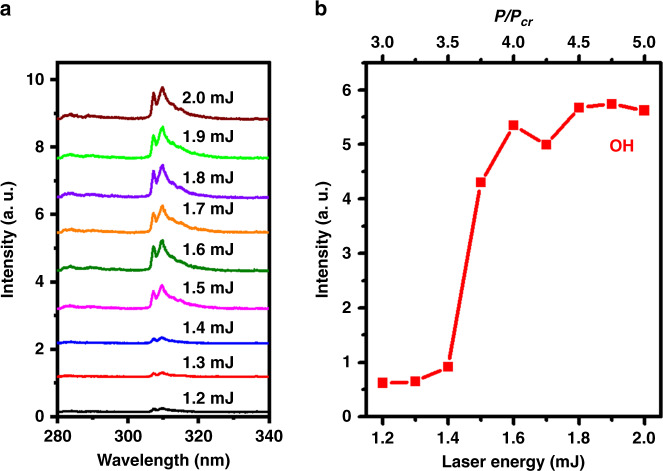
OH radical fluorescence measurement. **a** Emission spectra at 50 ns after the laser arrives with a time integration of 100 μs in a CH_4_/air flow at different energies. **b** OH emission intensity with different incident laser energies

To determine the role of OH radicals in ultrashort LI, we also measured the OES spectra of OH radicals within an early time window (Δ*t* = 100 μs, and *t* = 50 ns) with different input laser energies, as shown in Fig. 5a. As the laser energy varies from 1.2 to 2.0 mJ, the spectral band of OH radicals at ~308.9 nm can be clearly observed for all cases, but their fluorescence intensities are significantly different. To clearly see the variation in OH fluorescence, Fig. 5b plots the OH fluorescence intensity integrated from 306.5 to 312.4 nm as a function of the input laser energy. It can be observed from Fig. 5b that the OH signal intensity remains almost the same when the laser energy is <1.4 mJ, but dramatically increases and then saturates as the laser energy increases from 1.4 to 2.0 mJ. The variation trend of OH fluorescence measured at early times with different energies agrees with the ignition results shown in Fig. 1b, and indicates direct evidence for the correlation between the OH radical number density and the ultimate LI. That is, when the number density of OH radicals reaches a certain level, a flame forms^31^. Therefore, OH radicals can serve as an indicator of laser filament ignition.

## Discussion

Based on previous measurements in air^32^, for a focused Ti:sapphire laser beam (800 nm and 40 fs) with a 20 cm focal length, the plasma density is estimated to be ~10^17^–10^18^ cm^−3^. The critical power, *P*_cr_, for Kerr self-focusing in air is ∼10 GW (ref. ^23^), and the clamped laser intensity is ~10^14^ W cm^−2^ (ref. ^33^). Because the volume fraction of air molecules in the mixture is ~92% and the first ionization energy (12.6 eV) of methane molecules lies between oxygen molecules (12.1 eV) and nitrogen molecules (15.6 eV)^34^, we can adopt these values to understand the current experimental results. As a result, when the laser energy varies from 1.2 to 2.0 mJ, the peak power of the incident laser pulse increases from ∼3 *P*_cr_ to 5 *P*_cr_, as shown in Figs. 4b and 5b.

The laser filament generated in the mixture establishes a high-density plasma pool, which consists of abundant ionized or neutrally fragmented products of combustible mixtures such as N_2_^+^, O_2_^−^, and O_3_, as well as hydrocarbon fragments. In particular, it is known that in high-density methane/air plasma, a large number of oxygen atoms can be formed by the following reactions^19^:

1 $${\mathrm{N}}^ + + {\mathrm{O}}_{\mathrm{2}} \to {\mathrm{NO}}^ + + {\mathrm{O}}$$

2 $${\mathrm{N}} + {\mathrm{O}}_{\mathrm{2}} \to {\mathrm{NO}} + {\mathrm{O}}$$

3 $${\mathrm{e}} + {\mathrm{O}}_{\mathrm{2}} \to {\mathrm{O}} + {\mathrm{O}}$$

Due to Raman excitation, ionization and fragmentation, some of the laser energy is coupled to the plasma, heating the combustible mixture and resulting in a gas temperature of ~1400 K, as reported in air^16^. In this temperature region, the temperature-sensitive hydrogen abstraction from the collision of CH_4_ molecules with atomic oxygen fragments becomes efficient (ref. ^22^), producing OH radicals through the reaction:

4 $${\mathrm{CH}}_{\mathrm{4}} + {\mathrm{O}} \to {\mathrm{CH}}_{\mathrm{3}} + {\mathrm{OH}}$$

Associating with OH radical formation, chain-branching oxidation reactions start, and finally, combustion occurs, resulting in a flame. It should be noted that there is another reaction routine producing OH radicals by methane pyrolysis^22^. However, this path is not efficient until the gas temperature exceeds 2500 K. Therefore, the contribution of thermal decomposition to producing OH radicals in ultrashort LI can be neglected.

We also consider the possible mechanisms responsible for the misfire and fire results shown in Fig. 1b. Since under all laser energy conditions, a laser filament is formed, the laser intensity and thus the initial temperature are considered the same for all cases. However, as the input laser energy increases, although the laser intensity in the filament is fixed, the plasma density and volume can still increase^32^, producing a larger OH radical number density early in the process, as shown in Fig. 5b. Additional OH radicals promote chain-propagating reactions, releasing more heat energy and speeding up the oxidation reactions of CH_4_ molecules. In addition, the extension of the filament length in the case of the high input laser energy enhances the line ignition effect, in favor of combustion development. However, it should also be noted that successful ignition by the laser filament requires a proper balance between the plasma density and the filament length, which are sensitive to external focusing conditions^32,33^.

The surge in OH fluorescence intensity from misfire to fire in filament-induced ignition is strikingly different from that in ns-LISI, where the signal intensity linearly increases as the laser energy increases around the threshold^25^. The significant increase in OH radicals around the ignition threshold in the filament case shows a clear boundary between successful and failed ignition events. However, in the ns-LISI scheme, OH radicals mainly come from atomic recombination in the cooling process of hot plasma generated by breakdown. The stochastic nature of the breakdown around the threshold affects chain branching, leading to potential ignition failure^29^. Since there is no breakdown in ultrashort LI, the ignition result can be readily predetermined.

In summary, we demonstrated that the ignition of a lean methane/air mixture can be unambiguously achieved with an extremely low sub-mJ minimum ignition energy and ultrahigh rate of success by an ultrashort fs pulsed laser that propagates in the filamentation regime. By a series of nonlinear effects, such as Raman excitation, strong-field ionization and molecular dissociation, the high-intensity laser filament establishes a high-density free radical pool with a gas temperature of ~1400 K in the combustible mixture. Many OH radicals produced in the high-temperature radical pool are essential for ultrashort LI. The robustness of this ignition scheme derives from the unique property of the laser filament, i.e., the sustained high level of optical plasma density inside the longitudinally extended filament core, giving rise to simultaneous ignition along the filament line. The present approach, in which the ultrashort LI of lean-fuel mixtures works in a relatively low-temperature and centimeter-long plasma filament, provides possibilities for investigating ultrafast physical/chemical processes on the fs/ps timescale after the laser–fuel interaction, and has general applicability to complex combustion conditions in a variety of engines that are not in stoichiometric ratios^35^.

## Methods

We carried out experiments with linearly polarized 800-nm and 40-fs laser pulses, which were produced from a Ti:sapphire femtosecond laser system (Spectra Physics, Spitfire ACE). The repetition rate ranged from 4 to 1 kHz, and the output energy was ~2.5 mJ, which could be attenuated by a half-wave plate and a polarizer. The laser pulse was focused by a fused silica lens (*f* = 20 cm) to generate a single filament located 10 mm above a McKenna burner, whose configuration can be found in ref. ^28^. The filament length was measured to be ~0.8 cm for an input laser energy of 2.0 mJ. The velocity of the premixed methane/air gas was set at 1 m s^−1^ with a Reynolds number of 670 so that the gas flow was laminar. The premixed laminar flow was set at a fuel-lean condition with an equivalence ratio of *φ* = 0.82 ± 0.02 for subsequent ignition experiments, in which flames that were not self-sustaining were studied.

For the spectral and imaging measurements, we collected the optical radiations emitted from the filament or the ignited flame in the direction perpendicular to the laser propagation by a fused silica lens (*f* = 6 cm), using a 7:1 telescope imaging scheme. The collected light was then focused on the entrance slit of a spectrometer (Andor Shamrock SR-750i) equipped with an ICCD camera (Andor iStar). For the spectral measurement, the slit width of the spectrometer was set at 200 μm, and the light was dispersed by a 500 lines mm^−1^ grating and then captured by the ICCD. The laser repetition rate was set at 100 Hz. For the imaging measurement, the slit width was adjusted to 2.5 mm, and the grating was changed to zero order so that images could be directly taken by the ICCD. The laser repetition rate was set at 4 Hz.

For monitoring the laser filament ignitions at different energies shown in Fig. 1b, the ICCD gate was opened with a time window of Δ*t* = 20 ms and a time delay of *t* = −5 ns. When measuring the flame kernel dynamics and time-resolved spectra shown in Figs. 2 and 3, the gate window of the ICCD was set to be Δ*t* = 50 μs, and the gate delay varied from *t* = −5 ns to *t* = 6 ms. Note that the laser pulse arrival time at the interaction zone is *t* = 0 ns. When recording the OES spectra in an early time window, as shown in Fig. 5, the ICCD was opened for a fixed period of Δ*t* = 100 μs with a time delay of *t* = 50 ns. It should be emphasized that single-shot fs LI was unambiguously achieved, but 20 and 200 independent ignition events were accumulated for each image and spectrum, respectively, to increase their signal-to-noise ratio.

In the energy deposition measurement shown in Fig. 4, the laser energy was measured by a laser power meter, which was placed at positions 20 cm away from the burner before and after the filament. The laser repetition rates were 50, 100, and 200 Hz. The energy deposited into the plasma was calculated by subtracting the transmitted energy (measured after the filament) from the incident laser energy.
